# Concurrent Hairy Cell Leukemia and Metastatic Merkel Cell Carcinoma

**DOI:** 10.1155/2018/1736854

**Published:** 2018-11-14

**Authors:** Ajay Prakash, Alhossain A. Khalafallah

**Affiliations:** ^1^Tasmanian Medical Laboratory, Launceston, Tasmania, Australia; ^2^Specialist Care Australia, Launceston, Tasmania, Australia; ^3^Faculty of Health Sciences, University of Tasmania, Australia; ^4^Menzies Institute for Medical Research, University of Tasmania, Launceston, Tasmania, Australia

## Abstract

Hairy cell leukemia (HCL) and Merkel cell carcinoma (MCC) are two rare malignancies with distinct cells of origin. HCL is a lymphoid malignancy of mature B cells, and MCC derives from neuroendocrine cell origin. HCL has a favorable prognosis with most patients achieving long-term remission and potential cure. In contrast, MCC is an aggressive malignancy affecting the skin and can metastasize quickly with a dismal prognosis. Immunocompromised patients, such as those with AIDS, posttransplant, and the elderly, have higher incidences than the general population, suggesting a possible immune mechanism. We report a case where a patient presented with HCL and metastatic MCC synchronously. This is the first reported case of these two rare malignancies occurring concurrently at initial presentation and may represent a role of immunosuppression in the pathogenesis of MCC.

## 1. Background

Merkel cell carcinoma (MCC) is a rare and highly aggressive cutaneous malignancy, also known as “primary neuroendocrine carcinoma of the skin.” MCC occurs most often on the sun-exposed face, head, and neck, with an annual incidence of 1200 new cases each year in the US, which is far less than other forms of skin cancers and has a 2 : 1 predominance in males [1]. The tumor tends to invade underlying subcutaneous fat, fascia, and muscle and spreads quickly to regional lymph nodes. Haematogenous metastasis is common to the liver, lungs, brain, and bone. Immune suppression greatly increases the risk of developing MCC, with the incidence 13.4 times more common in advanced HIV and similarly in solid organ transplant recipients [2, 3].

Hairy cell leukemia (HCL) is an uncommon haematological malignancy characterized by clonal disease of mature B lymphocytes with fine villous cytoplasmic projections (hence the name hairy cells) found in the bone marrow and peripheral blood. HCL represents approximately 2% of all lymphomas with an annual occurrence of approximately 3/100,000 with a median age over 50 years with male predominance of four to five times over females [4].

HCL is known to be associated with systemic immunologic disorders including scleroderma, polymyositis, polyarteritis nodosa, erythematous maculopapules, and pyoderma gangrenosum. Other known associations are acquired factor VIII antibodies, paraproteinaemia, and systemic mast cell disease.

The diagnostic investigations include bone marrow biopsy to confirm the presence of the typical hairy cells and to exclude other diagnoses, such as splenic marginal zone lymphoma or B-cell prolymphocytic leukemia. There is no readily apparent association between MCC and HCL other than the increased risk of MCC in immunosuppressed patients. We present the case of a patient, diagnosed with HCL, based upon the demonstration of morphological typical hairy cells in the peripheral blood and bone marrow, which was found to have extensive, concurrent bone marrow metastasis by MCC on trephine histology. This simultaneous occurrence of these two rare malignancies at initial presentation has not been previously reported.

## 2. Case Presentation

We present a 56-year-old gentleman who worked as a computer programmer, with no significant past medical history. The patient was referred to the haematology department by his general practitioner with pancytopenia and splenomegaly. He described progressive left leg swelling and abdominal discomfort over the last 2 weeks prior to presentation. He had an increasing dyspnoea for one week. The patient was admitted to the hospital with ongoing fever with temperature > 38 degree Celsius.

Family history revealed an increased incidence of malignancies. His father was diagnosed and was being treated for low-grade lymphoma at that time. His sister had a history of renal cell carcinoma and was in remission following nephrectomy.

**Figure 1:**
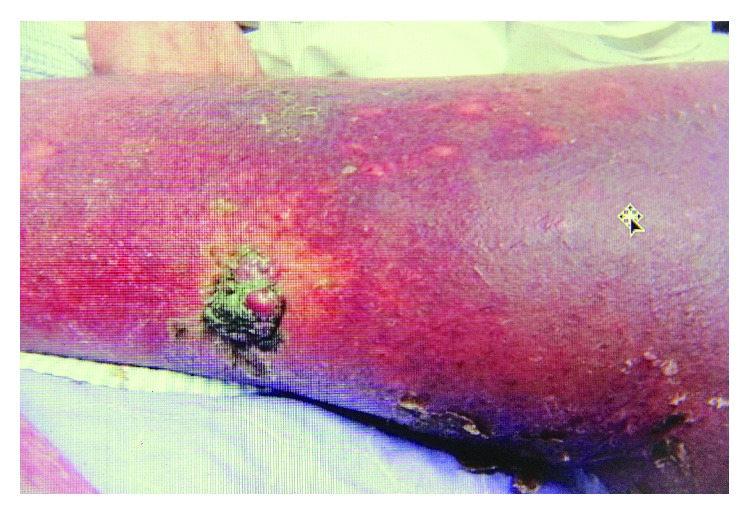
Merkel cell carcinoma skin lesion.

Clinical examination revealed marked splenomegaly (12 cm below the costal margin), markedly swollen left leg, and several small skin nodules over the anterior aspect of his leg (Figure 1).

Similar lesions were present on the right upper back. The left inguinal region was difficult to palpate due to oedema; however, there was no palpable lymphadenopathy elsewhere. There were petechial rashes on both lower limbs, being more extensive on the left side.

The initial investigations with peripheral blood smear and flow cytometry was suggestive of HCL. Staging CT scan showed lymphadenopathy in the para-aortic and iliac/inguinal region. Bone marrow biopsy confirmed concurrent HCL and MCC as described in Investigations. The biopsy from the skin lesion also confirmed MCC.

MCC has a poor overall prognosis, and confirmation of this diagnosis in this patient precluded him from treatment with curative intent.

### 2.1. Investigations

Initial investigations showed positive direct antiglobulin test (DAT) indicating autoimmune haemolytic anaemia with haemoglobin (Hb) of 50 g/L. The patient also had severe pancytopenia with a platelet count of 6 × 10^9^/L (N. 150-400 × 10^9^/L) and a white cell count of 0.4 × 10^9^/L (N. 4.0-11.0 × 10^9^/L). The LDH was elevated at 410 U/L (N. <250 U/L). The remaining serum chemistry values were within the normal reference range.

The peripheral blood film showed the presence of occasional hairy cells. Flow cytometry of the peripheral blood demonstrated a small clonal population of B lymphocytes expressing kappa-light chain restriction and the following cell surface immunophenotype: CD19+, CD20+, CD11c+, CD25+, CD103+, FMC7+, CD10−, and CD5−. This picture was consistent with HCL.

Staging CT scan showed enlarged para-aortic, left common iliac, left external iliac, left internal iliac, and left inguinal lymph nodes. The lumens of the left common iliac and external iliac veins were nearly completely obliterated by the adjacent enlarged lymph nodes; however, no thrombosis was noted.

Bone marrow biopsy revealed a hypercellular marrow with distortion of the normal marrow architecture by the presence of a diffuse lymphocytic infiltration, increased fibrosis, and areas replaced by large, immature, and undifferentiated malignant cells.

**Figure 2:**
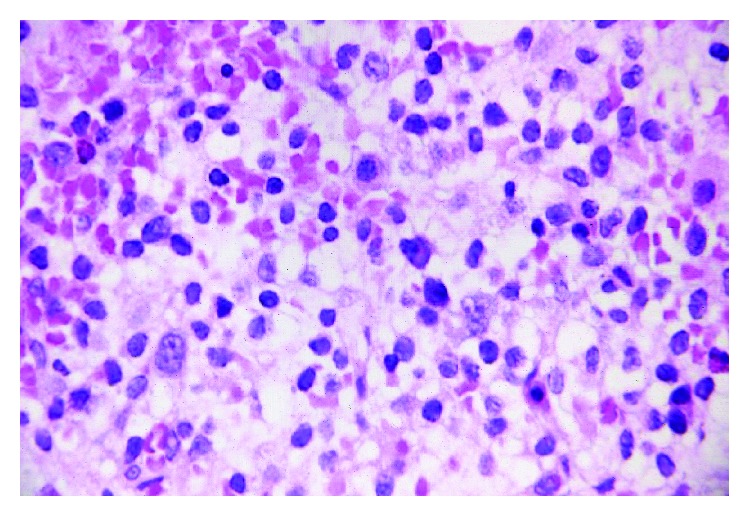
Fried egg appearance; bone marrow trephine (H&E stain).

**Figure 3:**
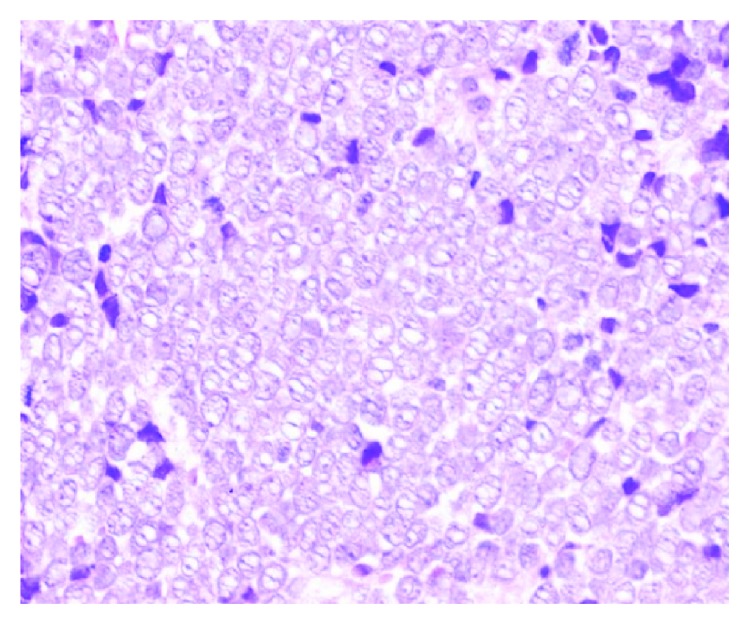
Merkel cell infiltration of bone marrow trephine (H&E stain).

**Figure 4:**
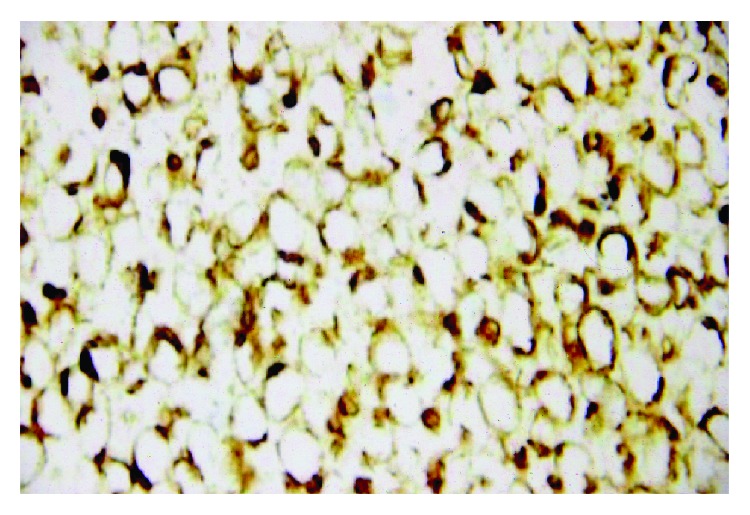
Chromogranin staining Merkel cells in BM trephine.

**Figure 5:**
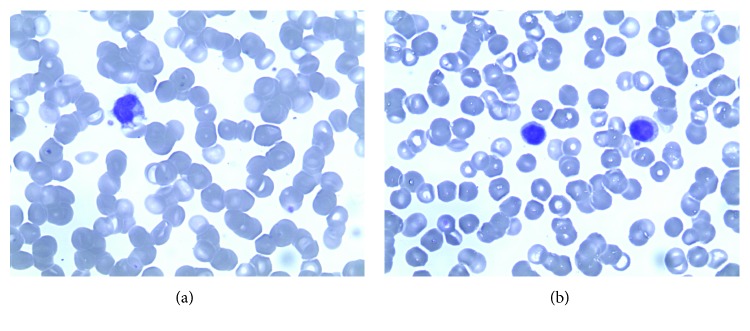
(a, b) Hairy cells in the peripheral blood H&E stain.

The bone marrow trephine demonstrated a “fried egg appearance,” a morphological feature considered highly suggestive of HCL (Figure 2). Bone marrow trephine also showed large nonhaematopoietic tumor cells appearing to grow in organoid formation suggestive of an undifferentiated carcinoma (Figure 3). They displayed conspicuous nucleoli and lamellar structures within the cytoplasm. Immunohistochemical stains were positive for cytokeratin-20 (CK-20), chromogranin A (Figure 4) and synaptophysin and negative for CD45, CD20, and cytokeratin-7 (CK-7). The characteristic cytoplasmic hairy projections of the lymphoid cells are demonstrated in Figures 5(a) and 5(b).

Subsequent biopsy of the skin lesions demonstrated dermal infiltration by “small round blue cells.” These neoplastic cells formed nests and sheets that insinuated between the dermal collagen bundles. The tumor cells had no appreciable cytoplasm, with round to irregular nuclei, fine granular chromatin, and strong positive labelling with the neuroendocrine marker synaptophysin. These cells stained positively for pancytokeratin (AE1/3) in a dot distribution and did not stain with lymphoid markers CD20 or CD3. The morphology was classic for MCC and similar in appearance to the nonhaematopoietic cell infiltrate present in bone marrow trephine.

### 2.2. Differential Diagnosis

1. metastatic visceral neuroendocrine carcinomas
2. cutaneous lymphoma
3. dermatologic manifestations of systemic immunologic disorders, like scleroderma, polymyositis, and polyarteritis nodosa
4. other skin malignancies, like squamous cell carcinoma
5. viral infection

### 2.3. Treatment

The patient was treated with weekly intravenous infusions of carboplatin, supported with granulocyte colony stimulating factor (G-CSF) and combined with pulsed high-dose steroids and intravenous immunoglobulin. The patient received the above therapy for approximately 6 weeks. Involved field radiotherapy to the left inguinal region was also administered in an effort to reduce the extent of left lower limb oedema and provide symptomatic relief.

### 2.4. Outcome and Follow-Up

There was some transient improvement in his neutrophil and red cell counts; however, he continued to be refractory to platelet transfusion. The symptoms in the left lower limb continued to progress with increasing oedema, despite involved field radiotherapy to the left inguinal region. As the symptoms continued to worsen despite treatment, it was decided to opt for palliation thereafter. Because of metastatic MCC and associated poor prognosis, it was decided not to commence the treatment for hairy cell leukemia.

The patient passed away after 8 weeks of palliation as a consequence of worsening cytopenias and systemic bacterial infection.

## 3. Discussion

Merkel cell carcinoma (MCC) is a rare neuroendocrine tumor that presents as a painless, flesh-colored, and red or blue skin nodular lesion that varies in size from 0.5 cm to more than 5 cm. Approximately half of all MCC occur on sun-exposed areas of the head and neck, one-third begin on the legs, and 15% occur on the arms and less commonly on other parts of the body such as the trunk. MCC most commonly affects Caucasians between ages of 60 and 80 years with a male predominance of 2 : 1 [5]. MCC is “a small round blue cell tumor” and must be differentiated from metastatic visceral neuroendocrine carcinomas, particularly from small cell lung carcinoma (SCLC) or other small round cell tumors [6]. This distinction is accomplished with near certainty using immunohistochemical analysis. CK-20 is a highly sensitive marker for MCC, staining positively in a paranuclear, dot-like pattern in 89% to 100% of tumors [7]. Thyroid transcription factor-1 (TTF-1) is expressed in 83% to 100% of SCLC yet is consistently absent in MCC [8, 9]. CK-7 is expressed in SCLC but is characteristically negative in MCC. Other markers with high sensitivity for MCC and to a lesser degree for SCLC include neuron-specific enolase, chromogranin A, synaptophysin, and BER-EP4 [8, 9]. MCC is invariably negative for S-100 and leukocyte-common antigen, distinguishing it from small cell melanoma and cutaneous lymphoma, respectively.

Hairy cell leukemia (HCL), on the other hand, is a chronic B-lymphoid leukemia that was originally described in 1958 by Bouroncle et al. and Cannon et al. and has characteristic villous-like cytoplasmic projections in the affected lymphocytes [10, 11].

HCL is more common in Caucasians and rare in those of Japanese and African origin [11]. HCL presents with a median age of 52 years and has a 4-5-fold male predominance. HCL infiltrates the reticuloendothelial system, spleen, liver and bone marrow, resulting in organomegaly, marrow failure, and pancytopenia. Generalized abdominal lymphadenopathy is not a routine feature of HCL. The etiology of HCL is unknown, although a French study that evaluated occupational exposure to pesticides and development of lymphoid neoplasms among men appears to support the hypothesis that occupational pesticide exposures may correlate with development of a number of lymphoid malignancies including hairy cell leukemia, Hodgkin lymphoma, multiple myeloma, and non-Hodgkin lymphoma [12].

Hairy cells can be identified immunophenotypically in 92% of cases, even when the cells represent less than 1% of the circulating lymphocytes. They have a mature B-cell phenotype and typically express single or multiple immunoglobulin light chains and pan-B-cell antigens, such as CD19, CD20, CD22, and CD79b, but not CD21 (late B-cell marker). The cells also typically express CD103, CD11c, and CD25 but usually not CD5, CD10, or CD23. As with the case presented here, the presence of features atypical for HCL, such as massive abdominal lymphadenopathy and skin lesions, prompted further investigations for additional diagnoses.

Standard treatment for HCL with 2-CDA or pentostatin can achieve long-term remission and excellent response that may last for several years [11]. Such treatment has the potential for improving cytopenia and the symptoms of splenomegaly [11].

A virus called Merkel cell polyomavirus (MCV) is suspected to contribute to the development of the majority of MCC. Approximately 80% of MCC have this virus integrated in a monoclonal pattern [12], indicating that the infection was present in a precursor cell prior to malignant transformation.

At least 20% of MCC tumors are not infected with MCV suggesting that MCC may have other etiologies. MCC occurs more frequently than expected among immunosuppressed patients, such as posttransplant, advanced HIV infection, the elderly, and patients receiving systemic immunosuppressive agents, suggesting an immunomodulatory mechanism [2, 3, 13]. The incidence of MCC is significantly increased in immunocompromised patients who undergo renal transplant and occurs several years posttransplant [14]. Most of these patients are relatively young, and the incidence of MCC follows an aggressive and often fatal course. There have been few case reports of MCC developing after many years in chronic lymphocytic leukemia (CLL) patients [15]. Furthermore, a large proportion of CLL patients demonstrate Merkel cell polyomavirus DNA positivity, suggesting sequential association between Merkel cell polyomavirus infection and immunosuppression for development of Merkel cell carcinoma [16]. The case presented here represents the first published report of MCC and HCL occurring simultaneously in the same patient. Further unusual features regarding this case are that the two rare malignancies were both present at the time of diagnosis and there was no antecedent history of drug- or disease-induced immunosuppression.

This patient had a strong family history of malignancy. Pathogenesis of HCL is poorly understood at the molecular level. However, recent studies have shown an important role for the heterozygous mutation V600E in the BRAF gene, which results in a variant protein with oncogenic function in HCL and other malignancies [17]. Patients with HCL may have increased incidence of second malignancies both before or after the diagnosis of HCL is made. The increased incidence is seen in both solid tumors (including skin cancers) and haematological malignancies. Furthermore, HCL may also be associated with familial malignancies [18].

HCL cells express phosphorylated mitogen-activated protein kinase (MEK) and extracellular-signal-regulated kinases (ERK), which are the downstream targets of the BRAF kinase, indicating constitutive activation of the RAF-MEK-ERK mitogen-activated protein kinase pathway in HCL. This finding may have implications for development of targeted therapies for HCL, in the future [17].

With current treatment options, MCC is often refractory and associated with a high incidence of recurrence and metastasis. It is hoped that an understanding of the molecular basis of this malignancy may lead to the development of antisense oligonucleotides and other targeted therapies. Mutational studies of platelet-derived growth factor receptor family of tyrosine kinases (PDGFRA) and tyrosine kinase receptor KIT (CD117) in Merkel cell carcinoma failed to demonstrate any activating mutations. Thus, targeted therapies with tyrosine kinase inhibitors like imatinib are unlikely to be effective [19]. Another study evaluated the expression of target molecules in Merkel cell carcinoma patients and reported the following: c-Kit (7%), myeloid cell leukemia sequence-1 (MCL-1) (88%), BMI-1 (78%), vascular endothelial growth factor-A (VEGF-A) (91%), VEGF-C (75%), VEGF-R2 (88%), PDGF-alpha (72%), and PDGF-beta (13%) [20]. There was no evidence of epidermal growth factor receptor (EGFR) and human epidermal growth factor receptor-2 (HER-2/neu) expression. High expression of BMI-1 and MCL-1 may need further studies as potential targets with antisense oligonucleotides in Merkel cell carcinoma.

This patient had a strong family history of malignancies in first-degree relatives with the father diagnosed with a low-grade non-Hodgkin's lymphoma (NHL) and the sister with renal cell carcinoma. It is likely that his family history predisposed him for HCL and the resultant immunosuppression led to development of MCC. However, it is possible that both HCL and MCC in this patient occurred due to hereditary predisposition.

Mutations in tumor suppressor and DNA repair genes can be inherited and predispose individuals to various malignancies [21]. Inherited malignancies are often multiple, present at a younger age, and are aggressive. TP53, CHEK2, STK11, PTEN, MSH2, APC, VHL, and BRCA1&2 are few of the genes which are implicated in hereditary predisposition for cancers. Merkel cell carcinoma has been rarely reported in association with hereditary cancer syndromes; however, the association and mechanism is still not clear. Merkel cell carcinoma has been reported in association with both renal cell carcinoma and Cowden's syndrome [22] and in association with Recklinghausen neurofibromatosis [23].

A recent study by Chan et al. suggested that the administration of immune checkpoint inhibition in MCC has the potential for future management strategy. This may integrate the use of a genetically modified chimeric antigen receptor T cell (CAR-T) therapy that targets the oncogenic Merkel cell polyomavirus (MCPyV) in both positive and negative MCPyV Merkel cell tumors [24].

Several clinical trials resulted in inclusion of avelumab, pembrolizumab, and nivolumab in the 2018 National Comprehensive Cancer Network (NCCN) guidelines as recommended treatment strategy for patients with disseminated MCC [25].

Further research into the molecular basis of inherited predisposition to cancer may facilitate in the development of targeted therapies.

### 3.1. Take Home Messages

1. Dermatologic signs in HCL patients should always be investigated further due to HCL's association with other immunologic and malignant disorders
2. MCC remains a difficult to treat condition, and more research is needed to better understand the etiology and to develop targeted therapies
3. Immunosuppression linked with relatively benign lymphoproliferative disorders may result in aggressive malignant conditions shifting the prognosis from good to poor
